# *Mycobacterium avium* Subsp. *avium* Infection in Four Veal Calves: Differentiation from Intestinal Tuberculosis

**DOI:** 10.1155/2014/715841

**Published:** 2014-02-13

**Authors:** Christine Goepfert, Nadine Regenscheit, Vanessa Schumacher, Simone Roos, Christophe Rossier, Corinne Baehler, Sarah Schmitt, Horst Posthaus

**Affiliations:** ^1^Institute of Animal Pathology, Vetsuisse Faculty, University of Bern, Länggassstraße 122, 3012 Bern, Switzerland; ^2^Kaelberpraxis, 6221 Rickenbach, Switzerland; ^3^Institute of Veterinary Bacteriology, Vetsuisse Faculty, University of Zurich, 8057 Zurich, Switzerland

## Abstract

*Mycobacterium avium* subsp. *avium* (Maa) is an intracellular pathogen belonging to the *Mycobacterium avium-intracellulare* complex (MAC). Reservoirs of MAC are the natural environment, wildlife and domestic animals. In adult bovine, MAC infections are typically caused by *Mycobacterium avium* subsp. *paratuberculosis* (Map). Maa infections in bovine are rarely reported but may cause clinical disease and pathological lesions similar to those observed in paratuberculosis or those induced by members of the *Mycobacterium tuberculosis* complex (MTBC). Therefore, differentiation of MAC from MTBC infection should be attempted, especially if unusual mycobacterial lesions are encountered. Four veal calves from a fattening farm dying with clinical signs of otitis media, fever, and weight loss were submitted for necropsy. Samples from affected organs were taken for histologic investigation, bacteriologic culture, and bacterial specification using PCR. Macroscopic thickening of the intestinal mucosa was induced by granulomatous enteritis and colitis. Intracytoplasmic acid-fast bacteria were detected by Ziehl-Neelsen stains and PCR revealed positive results for *Mycobacterium avium* subsp. *avium*. Clinical and pathological changes of Maa infection in veal calves had features of *Mycobacterium avium* subsp. *paratuberculosis* and the MTBC. Therefore, *Mycobacterium tuberculosis* complex infection should be considered in cases of granulomatous enteritis in calves.

## 1. Introduction

*Mycobacterium *(*M.*)* tuberculosis* and *M. bovis* are two important pathogens belonging to the *M. tuberculosis *complex (MTBC) causing human and bovine tuberculosis. Tuberculosis occurs spontaneously in cattle in developed countries and is endemic in Africa, Asia, and Central and South America with a herd infection rate of 10–35% [1]. Horses, small ruminants, swine, dogs, and cats can also be affected [1, 2]. Typical lesions are caseous granulomas mainly affecting the respiratory tract. In addition, enteric forms of tuberculosis with granulomatous changes and thickening of the intestinal mucosa also occur [1]. To prevent spread via the food chain, MTBC infections in food producing animals are classified as reportable diseases in many countries, and suspicious lesions in food animals must be investigated for the etiologic agent.

Nontuberculous bacteria of the *Mycobacterium avium-intracellulare *complex (MAC) most commonly cause enteric disease in animals [3]. Important members of this group are *M. avium* subsp. *paratuberculosis* (Map), the pathogenic agent of Johne's disease in ruminants that has also been isolated from human Crohn's disease patients [4], and *M. avium* subsp. *avium* (Maa). Maa is a primary pathogen in wild and domestic birds; mammals are however sporadically affected [5]. In humans, bacteria have been isolated from healthy individuals [6], but clinical disease may develop with immunosuppression [7]. MAC are widely distributed in the environment and have been isolated from soil, wastewater, water tanks, municipal water, aerosols, protozoa, deep litter, fresh tropical vegetation, animals, and humans [8]. Typically, MAC infections are characterized by granulomatous enteric lesions and intestinal lymphadenitis, but systemic disease can also occur [1]. Granulomatous lesions in the intestine of adult bovine are typically caused by Map (paratuberculosis), but this disease is usually not encountered in calves [9]. Maa induced lesions in calves have mainly been reported in experimental infections and are an unusual differential diagnosis of enteritis in veal calves [10]. Because of the similarity of enteric lesions induced by MTBC, differentiation of unusual mycobacterial enteric lesions in animals requires identification of the causative agent.

In the present case report we describe pathological and microbiological findings in four severely affected calves with granulomatous lesions in the intestine and mesenteric lymph nodes similar to those seen in Map. Due to the young age of the animals and the paucity of bacteria in the lesion, an infection with Map was however unlikely and MTBC as an infectious agent could not be excluded. Bacterial specification using PCR revealed positive results for Maa, which so far has only rarely been reported as enteric pathogen in calves.

## 2. Materials and Methods

### 2.1. Animals and Antemortem Evaluation

Calves bought at 7 to 34 days of age from different farms in western and central Switzerland were fattened for approximately five months. During the first five to six weeks of the fattening period, they were initially housed in groups of five and later on in groups of 45 calves on deep litter with open-air area. Within the last 9 weeks before death, three calves were housed on deep litter without open-air area in groups of 26, and one calf was housed in a group of 44 calves in a pen with open-air area. Medical attendance has been taking place regularly by a private veterinary clinic (Kaelberpraxis, Rickenbach, Switzerland).

### 2.2. Clinical Signs

Upon arrival to the fattening farm, calves weighed between 43 and 69 kg. On day one every calf was vaccinated against bovine respiratory syncytial virus (BRSV) and parainfluenza-3 virus (PI-3) and received Vitamin E, selenium, and iron per os or by injection. The prophylactic antibiotic applications were either SK60 (chlortetracycline and spiramycin) or Amoxan 70 (amoxicillin) 10 days per os combined with an injection of Draxxin 10% (tulathromycin). Shortly after arrival one calf showed colic signs, which was treated with Buscopan (butylscopolamine) and ColoSan (Sterculiae gummi) per os. For reasons of otitis media, respiratory symptoms, and fever, the affected fattening groups including each of the presented calves were treated once or several times with antibiotics, depending on the severity of the symptoms (CAS 45K, Primadox 50, SK60, or Amoxan 70). Over time three of the four calves were treated individually with antibiotics and anti-inflammatory drugs because of clinical signs of otitis media and elevated temperature. After six weeks of fattening, calves weighed between 74 and 103 kg, and after two to four months one calf was euthanized due to poor health (otitis media, apathy, and fever) and three calves died without apparent clinical signs. All four animals were submitted for necropsy.

### 2.3. Postmortem Examination

Dead calves were transported to the Institute of Animal Pathology, Vetsuisse Faculty, University of Bern, where a complete necropsy was performed. Samples from major organs were fixed in 4% neutral buffered formalin, routinely processed for paraffin embedding, sectioned (4 *μ*m), and stained with hematoxylin and eosin (H&E). Additionally, sections of mesenteric lymph nodes and intestine were stained with Ziehl-Neelsen (ZN) acid-fast stain.

Microscopically, lesions were graded as nonaffected (−), mildly (+), moderately (++), or severely (+++) affected as previously described [11]. Numbers of acid-fast bacilli were scored − (no acid-fast organisms), + (1–5 acid-fast bacilli/10 400x fields), ++ (6–50 acid-fast bacilli/10 400x fields), and +++ (>50 bacilli/10 400x fields).

### 2.4. PCR

DNA from fresh tissue (small and large intestine) of two calves was extracted using “QIAamp cador Pathogen Mini Kit” (Qiagen, Hilden, Germany) and subjected to specific real-time PCRs for detection of mycobacteria of the *Mycobacterium tuberculosis *complex (artus *M. tuberculosis* TM PCR Kit, Qiagen) and *Mycobacterium avium* subsp. *paratuberculosis* (TaqVet *Mycobacterium paratuberculosis *Advanced Real-Time PCR Kit, LSI, Lissieu, France). Additionally, sequencing of the 16 S rDNA was performed as previously described [12].

## 3. Results

### 3.1. Macroscopical Findings

**Figure 1:**
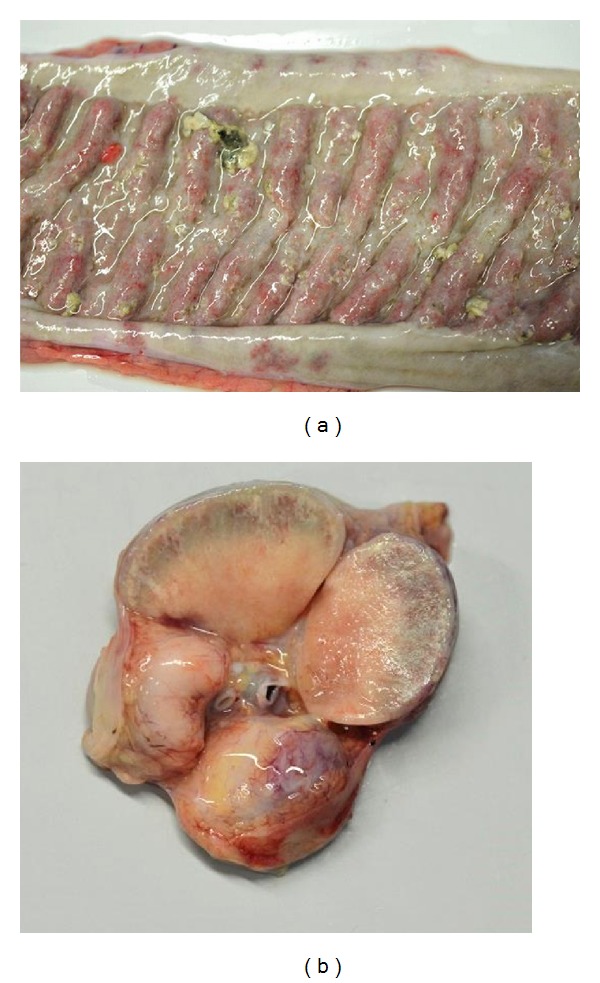
Macroscopic changes of intestine and lymph nodes. (a) Diffuse thickening of the ileal mucosa. (b) Lymphadenomegaly of ileocaecal lymph node.

Macroscopic changes were present within the small (jejunum and ileum) and large intestine (caecum and colon) and adjacent mesenteric lymph nodes. There was mild to severe segmental thickening of the intestinal mucosa, in severe cases with prominent horizontal folds (Figure 1(a)). The thickened mucosa was hyperaemic, and in three calves it contained multiple nodules of about 3 mm in diameter, which were ulcerated and filled with necrotic material. The intestinal contents were green and slurry and became pasty in the colon and rectum. Jejunal and ileocaecal lymph nodes were enlarged and measured up to 10 cm in diameter. External and cut surfaces were evenly pale and contained multiple caseous or mineralized areas of few millimetres in one calf (Figure 1(b)). Cranioventral areas of both lungs of two calves were firm and dark red and on the cut surfaces dry, white, and sometimes filled with pus. The pleura was multifocally roughened and covered with fibrin. Secretion residue was present on the fur of the ears in one calf.

### 3.2. Microscopical Lesions

**Figure 2:**
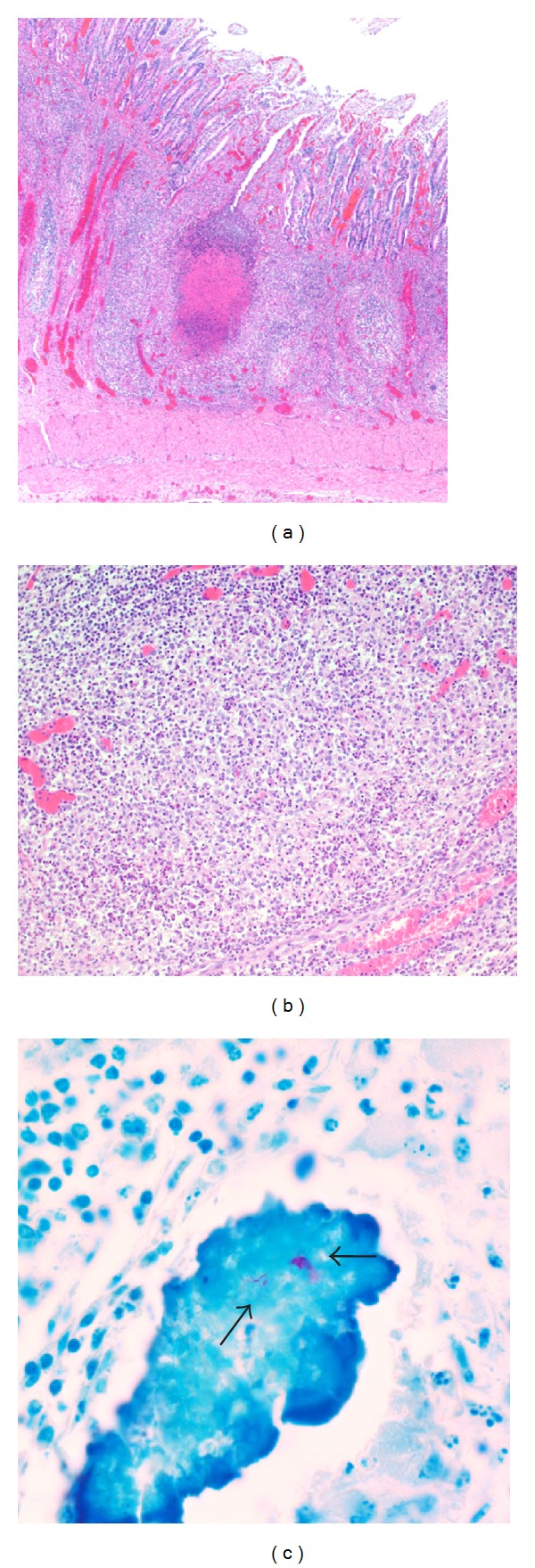
Histologic changes of intestine and lymph nodes: (a) pyogranulomatous enteritis (H&E stain, magnification 40x). (b) Higher magnification of A (H&E, magnification 200x). (c) Mesenteric lymph node. Granuloma with central calcification and intralesional, acid-fast bacilli (arrows, Ziehl-Neelsen stain, magnification 1000x).

The main histological findings of the small and large intestine are summarized in Table 1. There were mild to severe, multifocal infiltrates of macrophages and neutrophils in mucosa and submucosa. Macrophages were often loosely arranged in aggregates. Villi in the affected areas were shortened or fused, and there were numerous ulcers and necrotic foci (Figure 2(a)). Additional small aggregates of lymphocytes, plasma cells, and eosinophilic granulocytes were present in mucosa and submucosa. In the cortex of affected lymph nodes multiple areas of granulomatous inflammation containing multinucleated giant cells, macrophages, and central necrosis or mineralization were evident. Ziehl-Neelsen stains revealed intracytoplasmic acid-fast bacteria in macrophages and multinucleated giant cells (Figure 2(c)), which were only rarely observed in some affected areas of two calves (+). A low to moderate amount (+/++) of acid-fast bacilli was detected in affected tissues of the other two calves (Table 1).

In two animals the liver showed random necrotic foci, which were infiltrated by macrophages and neutrophils. One calf showed similar lesions in the spleen. The lung of two calves affected by bronchopneumonia showed severe, acute to chronic, necrosuppurative bronchitis/bronchiolitis and a fibrinous pleuritis. No acid-fast organisms were found from liver, spleen, or lung.

### 3.3. Bacteriology

Bacterial cultures of the intestine were negative in three calves, and in one calf high (small intestine)/moderate (large intestine) amounts of *Escherichia coli* type F41 were isolated. *Mycoplasma bovis* and *Bibersteinia trehalosi* were isolated from the lung of one calf and *Pasteurella multocida* subsp. *multocida/septica* from the second calf affected by bronchopneumonia.

### 3.4. PCR

Real-time PCR for detection of DNA of *Mycobacterium tuberculosis *complex and *Mycobacterium avium* subsp. *paratuberculosis *in intestinal and lymph node samples of two calves was negative.

Forward and reverse sequencing of the 16 S rDNA [12] followed by sequence comparison to the BLAST database revealed a sequence similarity of 99% for *Mycobacterium avium *subsp.* avium*.

Final diagnoses for the four calves were multifocal to coalescing, severe, and granulomatous enteritis and colitis. Additionally, two calves presented severe, acute/chronic active, necrosuppurative bronchopneumonia and fibrinous pleuritis, and one of those showed bilateral, acute, moderate to severe, purulent otitis media.

## 4. Discussion

Here, we report on four veal calves suffering from an unusual form of intestinal mycobacteriosis due to infection with Maa. Clinical signs were symptoms of otitis media, fever, and chronic weight loss, which represent common clinical complaints in veal calves. Bovines are prone to be infected by Map. Infection can take place under 30 days of age, but clinical disease does not develop until 2–5 years of age [9]. In contrast, disease in these calves already developed at 1.5 to 2 months of age. Macroscopic changes in paratuberculosis are segmental thickening of the ileum, caecum, and proximal colon with multifocal ulceration due to granulomatous inflammation and usually numerous intracytoplasmic acid-fast bacteria in macrophages [13]. In the reported cases, similar lesions were present in the intestinal tract; however, the macroscopic and histopathological appearance of ulcerative and necrotizing lesions as well as the various amounts (moderate to small amounts) of acid-fast bacilli warranted differentiation of other mycobacteria. In particular the observation of small numbers of intralesional acid-fast bacilli can be suspicious for MTBC infection [14]. MTBC in bovine is a reportable disease, and entrance of zoonotic bacteria into the food chain has to be avoided. Therefore, microbiological investigation at the National Reference Laboratory for mycobacteria was initiated. Final diagnosis of Maa as the etiologic agent was achieved by PCR and 16 S rDNA sequencing, thereby excluding *Mycobacterium tuberculosis* complex infection in these veal calves. Because Maa infections in animals are not reportable in Switzerland, no further action was mandatory at the veal calf operation. Nevertheless, remaining animals in the affected groups were closely monitored for signs of diarrhoea or weight loss. After slaughter of the remaining calves 5 months later and at approximately 230 kg no intestinal lesions or enlarged lymph nodes were reported at meat inspection. The source of infection of these calves remained unknown. Maa are widely distributed throughout the environment and it is likely that most calves housed in this goup were exposed. The affected calves additionally had other diseases during the fattening period, such as bacterial bronchopneumonia or otitis, and thus might have been predisposed to develop additional enteric mycobacteriosis. In humans it is well documented that Maa infection mainly occurs in immunocompromised individuals [15]. However, mycobacteriosis in these calves may also have been predisposed to secondary infections.

In conclusion, Maa infection in young calves can mimic clinical and pathological signs of paratuberculosis and intestinal tuberculosis. Because the disease is rarely reported as a cause of diarrhoea in calves, enteric mycobacterioses in this age group might be underdiagnosed. In addition, affected animals are expected to shed high numbers of Maa within the feces. In order to differentiate Maa lesions from those of MTBC and to reduce the distribution of bacteria in food animals such as calves, it is important to identify suspicious animals and initiate molecular testing of affected tissues.

## Figures and Tables

**Table 1:** 

Calf number	Granulomatous inflammation*					Necrosis*					Acid-fast organisms**				
	Jejunum	Ileum	Caecum	Colon	Lnn.	Jejunum	Ileum	Caecum	Colon	Lnn.	Jejunum	Ileum	Caecum	Colon	Lnn.
1	−	++	+	++	−	−	+	+	++	−	−	++	−	++	−
2	+	+++	−	−	+	−	++	−	−	+++	−	+	−	−	+
3	+	−	−	+	++	++	−	+	+	+	−	−	−	−	+
4	+++	+++	+++	++	+++	+++	+++	+++	+++	+++	+	++	++	+	+

*+: mild change; ++: moderate change; +++: severe change; −: negative.

**+: low number of acid-fast organisms; ++: moderate number of acid-fast organisms; +++: high number of acid-fast organisms.

Lnn.: Ileocaecal lymph node.
